# Time for prevention: workforce and population management approaches to implementing high-value preventive services in primary care

**DOI:** 10.1186/1748-5908-10-S1-A56

**Published:** 2015-08-20

**Authors:** Karin Johnson, Judith Schaefer, Lisa LeRoy, Kristin Mikolowsky, Kris Hansen

**Affiliations:** 1 Group Health Research Institute, Seattle, WA, 98101, USA; 2 Abt Associates, Cambridge, MA, 02138, USA

## Introduction

Getting the right preventive services to the right people at the right time is an enduring challenge in U.S. primary health care. Many people, especially the poor and ethnic or racial minorities, aren't getting the services they need. At the same time, other people get services that have no benefit or even cause harm. Primary care practices struggle with providing preventive services because of time constraints, conflicting priorities, communication problems, and lack of systems to facilitate delivery. This study examines the workforce and population management approaches that exemplary primary care practices use to implement evidence-based preventive care.

## Methods

We conducted site visits to nine exemplary primary care practices that are part of the Robert Wood Johnson Foundation's Learning from Effective Ambulatory Practices learning community. By shadowing the care team and interviewing clinicians and staff members, we gathered data on how practices use team structures, electronic health records and care management procedures to implement the 15 high-value adult clinical preventive services recommended by the U.S. Preventive Services Task Force.

How the research advances the field of D&I: We observed a dynamic process of providers dividing responsibilities with other team members, particularly medical assistants, in order to weave clinical preventive services into their day-to-day responsibilities. Electronic health records provide a critical but still developing role in not only identifying patients due for preventive services, but also supporting communication amongst the clinical team and between the team and patients around clinical preventive services. This study provides empirical insights about the organizational factors, including workforce and technology, that shape how evidence moves into practice in primary care.

Funded by: The Agency for Healthcare Research and Quality, Department of Health and Human Services, contract #HHSA290-2010-00004i, Task Order #3, and by the Robert Wood Johnson Foundation.

